# The contours of control

**DOI:** 10.1007/s11098-013-0236-1

**Published:** 2013-12-05

**Authors:** Joshua Shepherd

**Affiliations:** Oxford Centre for Neuroethics, University of Oxford, 16/17 St Ebbes Street, Littlegate House, Suite 8, Oxford, OX1 1PT UK

**Keywords:** Control, Philosophy of action, Non-deviant causation

## Abstract

Necessarily, if S lacks the ability to exercise (some degree of) control, S is not an agent. If S is not an agent, S cannot act intentionally, responsibly, or rationally, nor can S possess or exercise free will. In spite of the obvious importance of control, however, no general account of control exists. In this paper I reflect on the nature of control itself. I develop accounts of control’s exercise and control’s possession that illuminate what it is for degrees of control—that is, the degree of control an agent possesses or exercises in a given circumstance—to vary. Finally, I demonstrate the usefulness of the account on offer by showing how it generates a solution to a long-standing problem for causalist theories of action, namely, the problem of deviant causation.

## Introduction

Necessarily, if S lacks the ability to exercise (some degree of) control over anything—any state of affairs, event, process, object, or whatever—S is not an agent. If S is not an agent, S cannot act intentionally, responsibly, or rationally, nor can S possess or exercise free will. In spite of control’s importance, however, no general account of control exists. Typically one finds the theorist—on his or her way to more pressing matters—pausing to offer a characterization of a qualified *form* of control. Managerial control, ecological control, guidance control, plural voluntary control, executive control, conscious control, cognitive control, rational control, attentional control: the list goes on. To be sure, one finds insights in various regions of behavioral and cognitive science, and philosophy.^1^ These insights stem largely from reflection on *how* control might be implemented in a system like the human brain. Such reflection is useful, and work in this vein is influential in what follows. But the fact remains that one rarely finds anything approaching a rigorous discussion of control itself. In what follows, that is where I wish to direct attention.

The exercise of control is a straightforwardly causal phenomenon, and closely related to the notion of intentional action. Necessarily, if an agent J acts intentionally, J has exercised some degree of control in so doing. Standard causalist accounts of intentional action have it that an action is intentional only if that action is caused in an appropriate way by some relevant mental state (e.g., an intention) or its acquisition. Anti-causalists are fond of noting that in spite of repeated attempts, no satisfactory account of appropriate (or non-deviant) causation exists.^2^ One might blame these failures on our current lack of an account of control. After all, as Markus Schlosser has put it, “In all cases of deviance, some control-undermining state or event occurs between the agent’s reason states and an event produced by that agent” (2007, p. 188). A philosopher wants to distract a commentator, and intends to knock over a glass. But this intention upsets him such that his hand shakes uncontrollably and accidentally knocks the glass over (Mele 1992, p. 182). Due to the agent’s lack of control, the goal is accomplished deviantly. Given the close connection between control and non-deviant causation, we might expect a satisfying account of control to generate a satisfying account of non-deviant causation.

In this paper I develop an account of control that does just this. In Sect. 2 I discuss control’s exercise. In Sect. 3 I develop an account of control’s possession. In Sect. 4 I leverage this account to offer an account of non-deviant causation, as well as fix a problem with the explication of control’s exercise begun in Sect. 2.

## Control’s exercise

Agents exercise control in a wide variety of ways—e.g., when they order a coffee, play chess, swing a racquetball racket, imagine what their vacation will be like, or read a philosophy paper. In general, what is it agents do when they exercise control? As a first approximation, agents deploy behavior in the service of motivational states. In my view, a motivational state M can be served by controlled behavior if and only if (a) M represents events E or states of affairs S, and (b) M can play a non-deviant causal role in the production of (or, at minimum, attempts to produce) E or S. This restriction plausibly applies to desires as well as intentions, and perhaps to urges and beliefs about what ought to be done. In what follows, I focus on intentions. Intentions clearly meet the restriction, and serve as the paradigm motivational state in this context.^3^

When agents deploy behavior in service of an intention, they aim at success. Success involves a match between behavior and the representational content of the relevant intention. Consider a basketball player who intends to make a shot. We can stipulate that the representational content of the intention includes the following plan: ‘square to the basket, locate the rim, aim just beyond the front of the rim, follow through, make the shot.’^4^ When the agent is successful, she executes her intention as planned—she squares to the basket, locates the rim, aims just beyond the front of the rim, follows through, and makes the shot.

Talk of a content match between intention and behavior raises the following question: what is the representational content of an intention?^5^ In what follows I side with Alfred Mele (1992), who maintains that an intention’s representational content is a *plan*. “In the limiting case… one’s action plan is a representation of one’s performing a basic action of a certain type. In other cases, one’s plan is a representation of one’s prospective A-ing and of the route to A-ing that one intends to take” (p. 218). Mele leaves open the form a represented plan can take. I will too. It is plausible that plans can take as many forms as our representational capacities will allow (e.g. linguistic and otherwise conceptual, as well as imagistic and otherwise non-conceptual).

At the ground floor of agency, then, we have the capacity to form and commit to plans. What else does an agent need if she is to exercise control? One thing she needs is a kind of *causal potency*. Causal potency can be understood as those causal powers by which an agent behaves. Sometimes an agent’s behavior is caused by her mental states (or their neural realizers^6^), as in the case of intentional action. But sometimes an agent’s behavior bypasses mental states altogether, as in the case of brute reflex. The cases that interest us involve mental states. To a rough approximation, an agent’s exercise of causal potency can be measured in degrees, by indexing the exercise to a specific motivational state (in this case, to an intention). We can, for example, define approximation-level and perfect-level potency.

*Approximation*-*level Potency.* An agent J possesses approximation-level potency regarding intention I in circumstances C to degree D if and only if for J, I in C can play a causal role in the production of behavior that approximates I’s content to degree D.

*Perfect*-*level potency*. An agent J possesses perfect-level potency regarding intention I in circumstances C if and only if for J, I in C can play a causal role in the production of behavior that perfectly matches the content of I.

The possession of these forms of causal potency is not sufficient for the possession of corresponding forms of control. To see why, consider the following case.

*Batter.* Frankie stands in the batter’s box, trembling. Frankie tends to strike out, and he has never hit a home run before. Part of the problem is his swing: an ugly, spasmic motion that rarely approaches the ball. In batting practice, Frankie’s coach will put a ball on a tee in front of him. Frankie hits the tee more often than the ball. Even so, Frankie recently saw a film that convinced him one simply needs to believe in oneself. Thus convinced, Frankie eyes the pitcher and whispers to himself, ‘Just believe, Frankie!’ He then shuts his eyes and intends the following: ‘Swing hard, and hit a home run!’ Here comes the pitch. With eyes still closed, Frankie swings hard and connects, producing a long fly ball deep to left field that lands just beyond the fence.

In his specific circumstances, Frankie possesses perfect-level causal potency regarding his intention to hit a home run in the given circumstances. Even so, the home run does not constitute an exercise of control (even if the blind swing of the bat does, to some small degree).

What else does Frankie need? It is tempting to say that Frankie needs non-deviant causal links between intention and behavior. Frankie’s swing, which by stipulation was an ugly, spasmic thing, is analogous to the philosopher’s shaking hand. Both the swing and the hand happened to be in the right place at the right time, and as a result both played a deviant causal role in the achievement of the agent’s goal.

Giving into temptation gives us the beginnings of an account of control’s exercise that resembles causalist accounts of intentional action. Consider:

EC*. An agent J exercises control in service of an intention I to degree D if and only if J’s non-deviantly caused behavior approximates the representational content of I to degree D.

There is something right about EC*. It rules out cases like *Batter* as exercises of (high degrees of) control. Further, it is a very plausible idea that the degree of control an agent exercises has to do with the degree of approximation between behavior and representational content. An intention sometimes causes behavior that fails to perfectly follow the plan, and thus fails to perfectly match the content of the intention. Becky intends to make a shot that is “all net”—that goes in without hitting the rim or backboard. But the ball hits the front of the rim, then the backboard, and drops in. Clearly Becky exercised a degree of control—the shot was very close to all net, so close that it went in. But her behavior failed to perfectly match her intention. (If Becky bet money on making it all net, this failure will be important.) Assuming that the representational content of the intention is exactly the same, it seems Becky exercises less control regarding her intention if the shot is an inch shorter, hits the front rim and does not drop in, and even less if she shoots an airball.

Finally, EC* seems to capture a core truth about control’s exercise: the exercise of control is essentially a matter of an agent’s bringing behavior to match the content of a relevant intention (or more broadly, a relevant motivational state).

In spite of EC*’s admirable qualities, two worries linger. To make the first worry vivid, return to Becky and the basketball shot.^7^ Consider two cases in which the content of Becky’s intention is as follows: make a shot by placing the ball through the rim all net. In case 1, Becky’s shot smacks the back of the rim, but thanks to an odd bounce the ball grazes the top of the backboard and falls through. In case 2 Becky’s shot is *closer* to going in all net, but after hitting the rim a few times the shot rims out. Is it not true that in case 1 Becky exercises a greater degree of control—since the shot goes in—and does this not undermine the essential connection between control’s exercise and a match between behavior and content?

In fact, I think not. Consider why we want to award Becky a greater degree of control when the shot goes in: because she has accomplished (what was presumably) her main goal in shooting the ball. Approximating the content of an intention is good, but surely (at least in normal cases) agents bring behavior to match the content of their intentions *in order to accomplish a goal*. So a close approximation of behavior to content seems useless without goal accomplishment. True enough, but arguing that Becky exercises more control when the shot goes in ignores the deviant bounce that led to its doing so. We want an exercise of control to be a product of non-deviant causation: goal achievement on its own should not influence our judgment about an agent’s degree of control.

A second worry is more troubling. In short, EC*’s appeal to non-deviant causation is problematic. If there is no non-circular account of non-deviant causation in the offing, then we will rightly suspect that the account on offer is superficial. In effect, EC* will tell us that the exercise of control is essentially a matter of an agent’s bringing behavior to match the representational content of a relevant intention in a controlled way.

I think there is a solution to this problem. Since it stems from reflection on control’s possession, it will take another section before the solution comes into view.

## Control’s possession

Intuitively, an agent in possession of control in service of some intention is an agent possessed of a certain flexibility. An agent in control is poised to handle any number of extenuating circumstances as she brings behavior in line with the intention’s content. Recall Frankie’s lucky home run. One thing Frankie lacked was this flexibility—Frankie was not ready to handle any extenuating circumstances.

This flexibility is tied to another feature possessed by agents in control: the ability to *repeatedly* execute an intention. After we see Frankie hit the home run, we want to know whether he can do that again. It would be nice, of course, to see him do it again in very similar circumstances. But we might also wonder whether he can do that again in a flexible way. In general, then, we can say that an agent in possession of control in service of some intention is an agent prepared to repeatedly execute that intention, even in the face of extenuating circumstances.

To illustrate: hold fixed Frankie’s intention—since on the present analysis whatever degree of control Frankie possesses is control in service of that intention—and suppose a number of things: the ball comes in 1 mph faster or slower, or an inch higher or lower, or that Frankie’s muscles were slightly more fatigued, or that Frankie produced a slightly different arc of swing. We can vary Frankie’s circumstances any way we like and ask: across this set of circumstances, how frequently does Frankie evince the ability he evinced when he hit the home run? The answer to this question will give us a measure of the control Frankie possesses regarding his intention.

We should not overlook the following point. In order to make sense of what we might call flexible repeatability, we have to specify a certain set of circumstances. This is not necessarily to say that the possession of control is composed (even in part) of extrinsic properties. In discussing her view of causal powers, Rae Langton distinguishes between extrinsic properties and relational properties, as follows: “whether a property is *extrinsic* or *intrinsic* is primarily a metaphysical matter… whether a property is *relational* or *non*-*relational* is primarily a conceptual matter: it is relational just in case it can be represented only by a relational concept” (2006, p. 173). As Langton notes, it is natural to view causal powers as both intrinsic and relational: intrinsic because such powers are “compatible with loneliness” and relational because “we need to talk about other things when describing it” (p. 173). Plausibly the same is true of the control an agent possesses.

The control an agent possesses is plastic across circumstances. Agents lose limbs, muscle tissue, and brain cells. They also learn novel ways of performing tasks, and become adept with various tools. Here I agree with Andy Clark: “Advanced biological brains are by nature *open*-*ended opportunistic controllers*. Such controllers compute, pretty much on a moment-to-moment basis, what problem-solving resources are readily available and recruit them into temporary problem-solving wholes” (2007, p. 101). Given the ways circumstances impact the amount of control an agent possesses in service of an intention, the specification of a set of circumstances requires care.

Roughly, we can say that a set of circumstances is *well*-*selected* if and only if it enables an interesting measure of the control an agent possesses regarding an intention. Below I discuss various ways to accomplish this. But one obvious constraint on set selection is the following. The set should be *sufficiently large*—that is, large enough to generate robust statistical measures regarding whatever features of an agent or her environment are being manipulated across the set. Think of a set of circumstances with only two members: the case in which Frankie hits a home run, and a case in which he misses the ball. This set is not informative: we need a large number of cases before we get any useful information regarding just how lucky Frankie’s home run was. Another important constraint concerns an agent’s causal powers: clearly, in some circumstances an agent’s causal powers are enhanced (e.g., by tools or other performance enhancers), while in others an agent’s causal powers are diminished (e.g., by various forms of bodily or mental impairment). To get an interesting measure of the control an agent possesses regarding an intention, an agent’s causal powers must be fixed in some principled way: in Sects. 3.1–3.3 I discuss fruitful ways of doing this.

Recall that we are discussing control regarding an intention. And for any intention, we can specify a level of content approximation. Let us say that an agent J’s degree of repeatability DR regarding some level of content approximation L in a (well-selected) set of circumstances C is given by J’s success-rate at reaching L across C, where successes are exercises of causal potency to the relevant level of content approximation or higher. In my view, an agent’s degree of repeatability (regarding an intention) gives us the degree of control she possesses (regarding that intention). We can put this more formally as follows.

*PC*. J possesses control to degree DR regarding some level of content approximation L for an intention I if and only if J’s success-rate at reaching L is DR, where the success-rate is measured across a sufficiently large and well-selected set of counterfactual circumstances in which J possesses, and attempts to execute, I.

Perhaps an example will help. Bill is throwing darts. Across a set of 100 circumstances, Bill possesses the intention to hit a bullseye. Suppose, now, that Bill hits the bullseye 11 times: his success-rate at this “perfect” level of content-approximation is .11. We might focus on other levels of content-approximation as well. It is informative to know that Bill places the dart within one inch of the bullseye 46 times, and within five inches of the bullseye 82 times. We might even change the set of circumstances—adding in various challenging contingencies—in order to measure Bill’s control in various ways (I discuss such contingencies below). With each well-selected set of circumstances, and each level of content approximation, we learn a bit more about Bill’s control in service of the intention to hit the bullseye.

This raises an interesting question: is there anything that answers to what we might call Bill’s *total control* in service of an intention, or indeed, Bill’s total control over his behavior? Perhaps at any given time there is something that answers to Bill’s *total causal power*—but we have seen that causal power does not amount to control. Even so, we sometimes speak in ways that might be taken to connote something like an agent’s total control in some domain or other. We say things like, “As a dart player, Bill has much more control than Fred.” Or we say, “Bill has more control over his placement tonight than he did last Tuesday.” In my view, such talk should not be taken to refer to anything precise. Often such talk builds in assumptions about normal circumstances, or prototypical performances. Sometimes such talk involves useful oversimplification. Just as often I suspect such talk is unhelpfully vague. If we wish to be precise about the control an agent possesses (or exercises), I think the analyses offered above and below are the way forward. Our normal talk of control should be seen as a loose, sometimes helpful, sometimes confused, way of referring to the more precise understanding of control developed in this paper.

In the next three subsections, I discuss useful ways of selecting sets of scenarios.

### Hot and cold control

An agent’s degree of control across a wide range of circumstances is—assuming that the range of circumstances varies features of interest—an illuminating measure of expertise. In the 1997 NBA Finals—already a pressure packed situation that would unnerve most basketball players—Michael Jordan played game 5 with the flu. Although visibly weak, to the extent that he would lean on teammates during timeouts, Jordan scored 15 points in the fourth quarter and 38 points overall, including a game-winning three-point shot with 25 s left in the game. Among NBA fans at least, the game is now legend, referred to as ‘The Flu Game.’ It is legend because the expertise Jordan displayed is hard to fathom.

Jordan’s performance in The Flu Game is theoretically suggestive. In that game he faced two distinct sources of trouble, (a) difficult circumstances and (b) interfering motivation. Attention to both proves illuminating. I take interfering motivation first.

Let us say that interfering motivation exists regarding an agent J’s intention I at time t when (a) a motivational state S is occurrent at t in J and (b) S’s instantiating its functional role in normal agents and normal circumstances tends to undermine the successful execution of I. Whether a state counts as interfering thus depends on whether it is genuinely motivational. I will leave the notion intuitive: clearly desires, urges, and fears qualify. Such states dispose agents to act in certain ways—ways that can subvert occurrent intentions. Jim intends to lose 15 pounds in a month, but loses only 7. One reason Jim comes up short is that Jim loves high-calorie beer such as imperial stout, and his love of imperial stout gives rise to desires to drink it after dinner. Jim recognizes it is hard to lose 15 pounds in a month if one drinks too much imperial stout, but Jim’s desires are recalcitrant, and after short internal battles Jim often gives in to them. Importantly, the existence of interfering motivation need not actually interfere with an exercise of control. Jim’s wife Kim intends to lose 10 pounds in a month. Kim loves imperial stout too, and often desires to drink it after dinner. Kim, however, manages to deal with these desires, and partially as a result of this she loses 10 pounds.

What explains the difference between Kim and Jim? It is tempting to say that Jim’s desire to lose weight lacks sufficient motivational *strength*. Jim intends to lose 15 pounds, but—as athletes sometimes say—he doesn’t want it enough. In fact I think that motivational strength is an important factor, but that strength alone is insufficient.

To see why, consider the motivational strength of some motivational state M. How does a motivational state get its strength? Mele (2003, chap. 1) points out a few plausible sources. One source of strength comes from related motivational states. So the motivational strength of Jim’s intention to lose 15 pounds is strengthened by his desire to look slim and his desire to honor his original intention. And the strength of Jim’s intention is weakened by his desire to drink imperial stout and his desire to eat Chinese food. Another source of strength comes from the mode of representation of any of the motivational states that contribute to M’s strength. Noticing the familiar desire to drink stout, Jim may picture a glass of stout as a glass of oil. Doing so can influence the desire’s contribution to the strength of Jim’s intention. A third source of strength comes from cognitive states related to M. The psychologist George Ainslie has shown that desires for perceived rewards increase in strength as the reward approaches in time (Ainslie 2001). Beliefs about the nearness of rewards, then, plausibly contribute to M’s motivational strength.

Since our understanding of the motivational system is incomplete, any discussion of a state’s motivational strength will be as well. We know enough to know that when interfering motivation exists, motivational strength is important. But as the following case illustrates, we need more than this notion alone to capture the role an agent’s motivational system plays in the possession of control.

*Free throw*. There is no time left on the clock. Nic stands at the free throw line alone. His team is down one, and he must make this free throw or face devastating defeat. The gym is full of screaming fans, and Nic is very nervous. Even though Nic is a 91 % free throw shooter, Nic’s team is more nervous than he. For Nic has missed his last three free throws, and last week he pulled the same stunt, missing two shots that would have won the game. As it happens, Nic misses the shot.

Although Nic fails to exercise a certain degree of control, it is odd to think that he does so because the motivational strength of his intention is insufficient. Plausibly Nic tried very hard to block out the crowd noise, to focus on the rim, to shoot with perfect form, and so on. The motivational strength of Nic’s intention to make the free throw is beside the point. What Nic needs in this circumstance is not just motivational strength, but the ability to calm his nerves, ignore his fears, and shoot his normal highly accurate shot.

A voluminous literature—the one on self-control—investigates the kind of ability Nic needs. For present purposes we want not to account for self-control, but to capture the relevance of interfering motivation to the possession of control. Let us distinguish, then, between *Hot* and *Cold PC*, where *Hot PC* covers circumstances which include interfering motivation, and *Cold PC* does not.

*Hot PC*. J possesses hot control to degree HC regarding some level of content approximation L for an intention I if and only if J’s success-rate at reaching L is HC, where the success-rate is measured across a sufficiently large and well-selected set of counterfactual circumstances in which (a) J possesses and attempts to execute I, and (b) interfering motivation exists.

*Cold PC*. J possesses hot control to degree HC regarding some level of content approximation L for an intention I if and only if J’s success-rate at reaching L is HC, where the success-rate is measured across a sufficiently large and well-selected set of counterfactual circumstances in which (a) J possesses and attempts to execute I, and (b) no interfering motivation exists.

Interfering motivation commonly frustrates agents. We tend to possess more control in service of our intentions in the absence of interfering motivation. The distinction between Hot and Cold PC allows us to capture this fact. This distinction thus isolates, as philosophers of action have done in other ways, interfering motivation as a source of theoretical and practical interest.

### Narrow and wide control

Nic (like Jordan) faces not only interfering motivation, but difficult circumstances. Nic is fatigued and his cognitive and perceptual capacities are strained. Nic’s inability (and Jordan’s ability) to exercise control in spite of these difficult circumstances suggests an illuminating distinction. Let us distinguish between *agential* circumstances and *non*-*agential* circumstances, where agential circumstances directly involve an agent’s physical and mental constitution,^8^ including her occurrent cognitive, perceptual and motivational states, and non-agential circumstances refer to those not just mentioned. Let us then distinguish between the possession of degrees of *narrow* and *wide* control.

*Narrow PC*. J possesses narrow control to degree NC regarding some level of content approximation L for an intention I if and only if J’s success-rate at reaching L across C is NC, where the success-rate is measured across a sufficiently large and well-selected set of counterfactual circumstances in which (a) J possesses and attempts to execute intention I, and (b) all non-agential circumstances remain constant.

*Wide PC*. J possesses wide control to degree WC regarding some level of content approximation L for an intention I if and only if J’s success-rate at reaching L across C is WC, where the success-rate is measured across a sufficiently large and well-selected set of counterfactual circumstances in which (a) J possesses and attempts to execute intention I, and (b) non-agential circumstances vary.

*Narrow PC* isolates a theoretically interesting circumstance-type. At minimum, we want to know whether J’s success-rate is simply a product of internal noise. That is, we want to know what behavior J is capable of controlling in virtue of only those features isolated by the term “agential circumstances.”

Consider the difference between an amateur dart player and a pro dart player. Suppose both form an intention with exactly the same content—‘hit the bullseye’—and both execute the intention, hitting the bullseye. Now fix all non-agential circumstances and give both agents the same intention across a set of different agential circumstances which exclude interfering motivation. From scenario to scenario, our agents will think different thoughts, will direct attention in different ways, will be more or less tired, and so on. Though these differences are slight, it is plausible that the pro will exhibit a higher degree of repeatability at hitting bullseye across these circumstances. If so, the pro possesses a higher degree of narrow control.

Varying external circumstances allows us to consider distinct properties relevant to control. Suppose the amateur is pretty good at his pub, with his darts, but that other pubs and other darts distract him, significantly lowering his degree of repeatability. The pro, however, has a high degree of repeatability no matter the venue, and with most brands of darts. This type of example illuminates a potentially useful measure of control’s possession, related to the *wideness* of the set of scenarios under consideration. In general if J can exercise control with a certain degree of repeatability across a wider range of scenarios than K, J is plausibly *better* at executing the kind of intention in question than K.

The distinctions between hot and cold control and narrow and wide control are orthogonal. Hot and wide circumstances mimic those of real world agents over time, while cold and narrow circumstances allow us to focus on features of agents that real world examples easily obscure. That they can be combined to suit certain theoretical purposes is an advantage of the framework on offer.^9^

### Control in service of a goal

Above I have focused primarily on control in service of intentions. But I do not want to leave the impression that control in service of intentions exhausts what can be said about control. Recall that originally I framed the issue in terms of motivational states. While intentions are paradigmatic, useful insights emerge from considering other motivational states as well. Consider the way that desires (or perhaps beliefs about what ought to be done, or belief-desire pairs) sometimes set *goals* for agents.

To see why we might want to consider control in service of a goal, consider two agents playing darts: one a recreational player (Torrey) and the other a professional (Bill). Both possess distal intentions with this content: ‘Hit a triple-twenty.’ Knowing only this much, we can predict that Bill and Torrey will differ mentally in at least one of two ways. First, as they step up to throw, they will likely form different proximal intentions. Due to the time spent honing his skills, Bill’s repertoire of plans for executing his distal intention is larger, more complex and subtle, and so on. The details need not concern us—perhaps they include Bill’s stance, the feel of Bill’s throwing arm, the grip position of his fingers, the direction of Bill’s attention. What is important for our purposes is that the type and availability of plans plausibly contributes to Bill’s comparatively higher degree of control across both narrow and wide sets of circumstances and, since Bill likely has more practice facing interfering motivation in dart-throwing contexts, in hot circumstances as well.

Second, a difference in representational capacities might lead to a difference between Bill and Torrey regarding the original distal intention. When Bill intends to hit a triple-twenty, he plausibly does so by way of a plan that is more subtle and detailed than Torrey’s. If so, the content of the very intention which centrally causes Bill’s control-constituting behavior differs from the content of Torrey’s similar intention. Thus a strict comparison of control in service of this intention is not desirable.

We can accommodate the difference between Bill and Torrey here by specifying a goal independent of the agent’s intentions: something they are both capable of desiring, perhaps, such as the goal to hit a triple-twenty. In so doing we index our measure of control to something different than an agent’s intention, but the result is salutary. It captures, for example, the way an agent’s representational capacities contribute to her control, and it allows a more flexible measure of control.

*GC.* An agent J possesses (wide, narrow, hot or cold) control to degree GC regarding a goal G if and only if J’s success-rate at achieving G is GC, where the success-rate is measured across a sufficiently large and well-selected set of counterfactual circumstances in which J tries to accomplish G.

## Non-deviant causation and control’s exercise reformulated

In this section I use the account of control’s possession developed above to generate an account of non-deviant causation. Before I offer the account, consideration of typical cases of deviant causation will prove useful.

*Basic deviance* occurs in between mental state and behavior. A philosopher wants to distract a commentator, and intends to knock over a glass. But this intention upsets him such that his hand shakes uncontrollably and accidentally knocks the glass over (Mele 1992, p. 182). Since the intention does not cause behavior *in the right way*, the philosopher does not intentionally knock the glass over.

*Consequential deviance* occurs after the behavior has begun, and before it is complete. Jones intends to kill K by shooting him. Jones misses by a mile, but the noise from his rifle scares a nearby herd of pigs that trample K (Davidson 1980, p. 78). The killing is unintentional because, again, it was deviantly caused.^10^

Many have proposed solutions to these types of deviance (e.g., Bishop 1989; Enç 2004; Schlosser 2007). None enjoy consensus (as Paul 2011 and Aguilar 2012, among others, have recently noted). This is not the place to review the history of attempts to solve the problem of deviant causation. It will suffice to note that a central problem with previously proffered solutions is precisely that they fail to deliver an understanding of the control agents exercise in acting intentionally. That the crux of the matter should revolve around control is not surprising. As Jesús Aguilar has noted, “what all cases of deviance exhibit is precisely the undermining of the possibility for action by the undermining of agential control. In turn, this explains why any plausible strategy to deal with the problem of deviance boils down to an effort to restore in one way or another agential control” (2012, p. 9).

In my view, we can leverage an understanding of control into an account of non-deviant causation by focusing on control’s possession. So long as there is a well-selected set across which an agent evinces a sufficiently high degree of control regarding a relevant intention, then (I claim) the normal execution of that intention in a token instance of that set qualifies as an instance of non-deviant causation. Importantly, the notion of normal here is statistical. What we want is to capture the causal pathway(s) operative as an agent goes about goes about successfully executing an intention in whatever circumstances confront her. This proposal is similar in spirit to Aguilar’s (2012). Aguilar appeals to the notion of *reliability* in developing a sufficient condition for non-deviant causation: according to Aguilar, the mechanism that produces an intentional action must be reliable (2012, p. 10). In my view this is on the right track. One might thus see what follows here as a way to flesh out the nature of the reliability Aguilar discusses (for qualifications, see footnote 11).^11^

Consider a golfer attempting to hit a golf ball into the middle of a fairway. There are a number of ways the golfer might hit the ball, such that it lands in the fairway: the ball might ricochet off of a tree, or the golfer might lose her grip on the club, somehow still connecting with the ball in just the right way, and so on. We want to exclude those cases in which the causal route to the ball’s landing in the fairway is deviant. The account of control’s possession regarding an intention gives us the resources to do so.

*Non*-*deviant causation*. An agent J’s behavior in service of an intention I is non-deviantly caused in some token circumstance T if and only if (a) J’s behavior in T reaches a level of content-approximation L (to the content of I) that is within a normal range for J, where the normal range is defined by J’s behavior across a sufficiently large and well-selected set of counterfactual circumstances C of which T is a member, and (b) J’s behavior in T is produced by way of those causal pathways normally responsible for J’s successes at reaching L across C.

We want to exclude deviantly caused successes. *Non*-*deviant causation* does so by way of two requirements. The relevant level of content-approximation—the level our agent actually achieves—must be within the normal range for a well-selected set of counterfactual circumstances. So this will be something the agent can do repeatedly. And the causal pathways operative in the actual case must fall within the normal range as well. So there is no room for a deviant causal pathway to operate surreptitiously.^12^

*Non*-*deviant causation* gives us the tools to explain why cases of basic and consequential deviance are deviant. In cases of basic deviance, behavior produced by way of the causal pathways operative in such cases will not, in general, tend to succeed. There is no well-selected and sufficiently large set of counterfactual circumstances for which the causal pathway in question falls in the normal range. The same is true in cases of consequential deviance. The events that occur in such cases are not events possessed of a high degree of repeatability. Thus, across any well-selected set of circumstances, agents will evince very low success-rates when producing behavior in such ways.

A few examples will help clarify the account’s commitments.^13^ Dwight normally misses free throws (although suppose he can hit some part of the rim at a much higher rate). Suppose Dwight is a 50 % free throw shooter. Suppose Dwight intends to make a free throw, attempts a free throw, hits the rim but misses, and that Dwight’s behavior in reaching this level of content-approximation L is produced by causal pathways normally responsible for L in similar circumstances. *Non*-*deviant causation* says that Dwight’s behavior was non-deviantly caused. But in missing the free throw, didn’t Dwight fail to exercise control? No: Dwight exercised a degree of control (and in so doing his behavior was non-deviantly caused). The shot headed in the direction of the basket, coming as close as the rim, after all.

But now suppose that Dwight attempts another free throw, and this time the shot goes in. And suppose that Dwight’s behavior is produced by way of causal pathways normally responsible for this level of content-approximation. Thus, on the account of control’s exercise offered thus far, Dwight exercises a greater degree of control: his behavior more closely matches the content of his intention to make the free throw. Given this, one might press the following problem for the account of control on offer: is it not plausible to think that the causal pathways in these two cases will be indistinguishable in any relevant sense? And if there is no relevant difference between the causal pathways, is it not problematic to hold that Dwight exercises more control in one of the cases?

There are two ways to respond here. First, I could deny that the causal pathways are indistinguishable. Although the naked eye might not be able to distinguish differences, it is plausible that at a more fine-grained level, important differences exist. Perhaps in the case of the miss Dwight’s attention was distracted slightly, or the ball rolled off his fingertips at a slightly awkward angle. (It is implausible, in my view, that Dwight intended only to make the free throw: his plan for doing so almost certainly included details such as maintaining attention at the rim and releasing the ball at a certain point and at a correct angle and so on.)

But suppose we stipulate that no such fine-grained differences exist. This is conceivable. If in one case Dwight makes the shot and in the other misses (due to some minor difference in execution), and if the causal pathways operative in both cases are indistinguishable in any relevant sense, then it seems the difference between Dwight’s make and Dwight’s miss is *due to noise*. And if that is so, then the proper response is to deny that Dwight exercised more control in making the shot than in missing it.

To deal with this worry, then, we need to append a principle to the account of control’s exercise.

*Anti*-*noise*. Any difference in degree of content-approximation that is due to noise does not make a difference to the degree of control’s exercise.^14^

What is it for a difference in content-approximation to be *due to noise*? We can clarify this notion by way of resources utilized in Sect. 3. Consider Dwight again. Focus on narrow and cold control, and consider two well-selected and sufficiently large sets of circumstances across which Dwight’s non-agential circumstances remain constant, and across which there is no interfering motivation. Suppose, further, that though these sets are not identical, they are the same in all control-relevant respects: the lighting is the same, the air temperature is the same, the air pressure in the ball is the same, and so on. Since these sets are not identical (and since, at any rate, we are making no assumptions about determinism here), it is plausible that Dwight’s success-rate at any level of content-approximation will vary slightly across the sets. This will be a difference due to noise.

*Non*-*deviant causation*, while formulated in terms of control, can be easily extended to the notion of intentional action. All we need to add is the requirement that the level of content approximation mentioned in clause (a) is a level sufficient for intentional action, given the activity in question. How are we supposed to understand sufficiency here? This will depend on the type of activity in question. It is a familiar point that for many action-types, the dividing line between intentional and non-intentional action is vague (Mele and Moser 1994). All we need here is the claim that there is some level of content match that is sufficient to make true the judgment that the action in question is intentional.

Finally, we are now in position to clean up the formulation of control’s exercise offered in Sect. 2. Recall that in that section, I had to rely on an unanalyzed notion of non-deviance to account for the exercise of control. I no longer have to do so.

*EC*. An agent J exercises control in service of an intention I to degree D in some token circumstance T if and only if (a) J’s behavior in T approximates the representational content of I to (at least) degree D, (b) J’s behavior in T is within a normal range for J, where the normal range is defined by J’s behavior across a sufficiently large and well-selected set of counterfactual circumstances C of which T is a member, (c) the causal pathway producing J’s behavior in T is among those normally responsible for producing J’s successes at reaching the level of content-approximation represented by D across C.

## Conclusion

A mature philosophy of action needs an account of control. The account developed above offers a clear way to conceptualize control, as well as a framework for thinking about those features of agents and environments that enhance or diminish the degrees of control agents exercise and possess. Its value is in part constituted by the light it sheds on this under-explored but central feature of agency. Further, this account generates a novel view of non-deviant causation. Since deviant causation is often thought to constitute a lingering problem for causal theories of action, the account of control on offer should be welcomed by causalists.

## Footnotes

1 Highlights include Christensen and Hooker (2001), Clark (2007), Clarke (2010), Dennett (1984), Frith et al. (2000), Hurley (2001), Ismael (2011), Jeannerod (1997), Mele (1992), Mossel (2005), Wolpert and Kawato (1998), and Wu (2011).

2 For treatments of non-deviant causation, see Bishop (1989), Enç (2004), and Schlosser (2007). For worries about such treatments, see Ginet (1990), Sehon (1997), and Tännsjö (2009).

3 Plausibly behavior that successfully serves other motivational states will be more proximally caused by intentions containing representational content similar to the original motivational state. How similar is an interesting question, but one I do not consider here.

4 The representational content can be rather more complex than that. The content might include, for example, motor imagery and sub-plans. While shooting a basketball, my intention has sometimes included something like the following content: ‘focus on the feel of the ball on the tip of the index finger.’

5 Delicate issues lurk here. Philosophers of action have disagreed, for example, over whether intentions necessarily include self-referential content. One might also stake out positions on differences in content between different types of intentions, e.g., between intentions directed at the future (distal intentions) and intentions directed at the present (proximal intentions) (see Pacherie 2008). Fortunately, we do not need a full account of an intention’s content in order to understand the notion of a match between behavior and content.

6 For purposes of readability, I omit reference to neural realizers in what follows.

7 In conversation and in comments on earlier drafts, more than one philosopher raised this worry.

8 In my view, it is plausible to extend an agent’s physical constitution (and perhaps her mental: see Clark 2008) to relevant tools in the agent’s environment.

9 At this point some readers may have the following worry. All of the above analyses of aspects of control hold fixed an intention and an agent’s attempt to execute it. But one might think that agents also possess and exercise control over the intentions they come to possess. In fact I do think agents possess and exercise this form of control, but that this phenomenon can be understood straightforwardly in terms of the above accounts of control’s exercise and possession. Notice that intention selection is precisely the task of forming and committing to one plan amongst many—the task, that is, of making a decision. If this task is itself initiated and guided by an intention, then it should be possible to extend the above accounts of control’s exercise and possession to an account of control over intention selection, by treating control over intention selection as control in service of an intention to decide what to do. On Alfred Mele’s widely adopted view practical decisions are intentional in part because of the causal work of a mental state extrinsic to the decision itself—an intention to decide what to do (Mele 2003, pp. 197–202). More specifically, Mele holds that an agent acquires an intention to decide what to do in response to uncertainty about what to do. The acquisition of this intention initiates a process of deliberation—a process that might be characterized as a search for reasons upon which to decide. Assuming agents do possess and exercise control over their decisions, then, it should be possible to develop an account of control over decisions by focusing on either (a) the control an agent exercises in deliberating, or (b) the control an agent exercises at the moment of intention formation. It is beyond the scope of this paper to say much more about control over decisions. I offer a much more involved discussion in Shepherd (n.d.).

10 A third type of deviance, tertiary deviance, concerns the history of the relevant mental state (see Mele 1987, p. 56). My account of non-deviant causation is not intended to solve tertiary deviance. Since my account of control is indexed to particular intentions, the history of the intention does not fall within its scope.

11 Since Aguilar’s discussion of reliability does not entail the view I offer, I do not know if Aguilar would agree with this. The view I offer is, after all, very closely tied to the account of control developed above. Further, Aguilar applies his discussion of reliability only to overt basic actions—the account developed here is developed independently of an account of intentional action, but is intended to apply to all intentional action-types (mental and overt). Finally, in my view Aguilar’s discussion does not place sufficient weight on the importance of circumstance to a proper understanding of reliability. Even so, I suspect that Aguilar and I are in substantial agreement.

12 Is it possible for an agent to act intentionally (i.e., by way of non-deviant causation) the first time she ever tries to perform an action of some type? One might think that since (in my view) repeatability is necessary for non-deviant causation, the possibility of such a case poses a problem. But recall that the set of circumstances against which repeatability is measured is *counterfactual*: so long as the agent would evince repeatability in similar circumstances, we have non-deviant causation.

13 Thanks to Al Mele for pressing me to clarify my view here.

14 Notice this means that degrees of control’s exercise will be more accurately given as a range, rather than a specific number.
